# Design and Synthesis of Pro-Apoptotic Compounds Inspired by Diatom Oxylipins

**DOI:** 10.3390/md11114527

**Published:** 2013-11-13

**Authors:** Giovanna Romano, Emiliano Manzo, Gian Luigi Russo, Giuliana d’Ippolito, Adele Cutignano, Maria Russo, Angelo Fontana

**Affiliations:** 1 Stazione Zoologica Anton Dohrn, Napoli 80121, Italy; 2 Institute of Biomolecular Chemistry, National Research Council, Pozzuoli 80078, Italy; E-Mails: emiliano.manzo@icb.cnr.it (E.M.); adele.cutignano@icb.cnr.it (A.C.); angelo.fontana@icb.cnr.it (A.F.); 3 Institute of Food Sciences, National Research Council, Avellino 83100, Italy; E-Mails: glrusso@isa.cnr.it (G.L.R.); mrusso@isa.cnr.it (M.R.)

**Keywords:** diatoms, oxylipins, chemical synthesis, bioactivity, apoptosis

## Abstract

Oxylipins are a large and diverse family of fatty acid derivatives exhibiting different levels of oxidation of the carbon chain. They are involved in many biological functions in mammals, plants and diatoms. In this last group of organisms, they are suggested to play a role in the reproductive failure of copepod predators, showing clear pro-apoptotic effects on newborn nauplii. In this work, these compounds were tested for the ability to induce mitotic arrest in sea urchin embryos. We show for the first time that oxylipins have an increased efficacy in their corresponding methylated form. Natural oxylipins were also used as an inspiration for the rational design and synthesis of stable chemical analogs with apoptotic activity against tumor cell lines. This approach led to the synthesis of the linear C15-ketol (**22**) that was shown to induce apoptosis in human leukemia U-937 cells. These results are proof of the concept of the use of eco-physiological considerations as a platform to guide the search for novel drug candidates.

## 1. Introduction

Diatoms are a widespread group of marine microalgae that play a pivotal role in primary production and biogeochemical cycles in marine ecosystems. They produce a plethora of bioactive oxylipins, a large and diverse family of secondary metabolites derived from the oxidation of polyunsaturated fatty acids. Most of these compounds arise from oxidation of eicosapentaenoic acid and unsaturated C16 fatty acids catalyzed by specific lipoxygenases (LOXs) with different regio- and stereo-selectivity [1,2]. The extreme variability of the downstream reactions adds great diversity to oxylipin structures that in diatoms include hydroxy-, keto-, epoxyhydroxy-acids and polyunsaturated aldehydes.

Oxylipins are common bioactive secondary metabolites present in mammals as well as in plants, mosses, algae, bacteria and fungi. In mammals, members of the family, such as prostanoids, leukotrienes and thromboxanes, are involved in inflammation and in numerous homeostatic biological functions and also modulate the expression of genes involved in DNA transcription and lipid metabolism [3,4]. In plants, C_18_-derived oxylipins mainly serve as signal molecules regulating developmental processes and are involved in plant stress responses and innate immunity [5,6,7]. In diatoms, oxylipins have been correlated to the antiproliferative effect of diatoms on predator reproduction [8,9], whereas, with the exception of few studies [10,11], their physiological role as signaling molecules remains largely unexplored. However, while many studies have been reported on the effect of polyunsaturated short-chained aldehydes [12,13], the biological effect of non-volatile oxylipins has been scarcely explored [2,12]. For instance, Fontana and co-workers showed that copepod nauplii hatched from eggs treated with a pool of oxylipins were seriously damaged and had a large proportion of apoptotic tissue, as revealed by terminal deoxynucleotidyl transferase (TdT) dUTP Nick-End Labeling (TUNEL) staining [2]. In the present study, we investigated the activity of different types of natural oxylipins on developmental processes in sea urchin embryos. The structures of these compounds were also used as an inspiration to design and synthesize a series of analogs with pro-apoptotic activity against a human leukemia-derived cell line.

## 2. Results and Discussion

### 2.1. Inhibition of Cell Division by Natural Oxylipins

**Figure 1:**
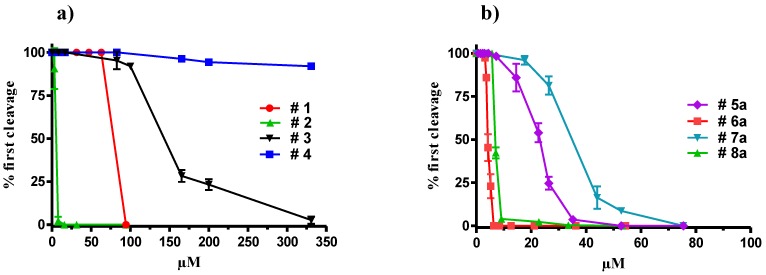
Percentage of first cleavage occurrence in sea urchin embryos treated with increasing concentrations of (**a**) 15*S*-hydroxyeicosapentaenoic acid (15(*S*)-HEPE) (#**1**), 15(*S*)-HEPE methyl ester (#**2**), eicosapenataenoic acid (EPA) (#**3**) and EPA methyl ester (#**4**) and of (**b**) natural oxylipins #**5a**–#**8a**. Compound numbering is according to the structure reported in Scheme 1. Values (means ± S.D.; *N* = 600) are the results of three different experiments.

15*S*-Hydroxyeicosapentaenoic acid (15(*S*)-HEPE) (**1**) is one of the most frequently encountered diatom oxylipins [13]. In agreement with previous studies on copepod nauplii reported in [12], this metabolite inhibited the cleavage in sea urchin *Paracentrotus lividus* embryos at concentrations higher than 70 µM and induced 100% blockage of cellular division at 94 µM (Figure 1a). Nevertheless, the methyl ester of 15(*S*)-HEPE (**2**) showed a noticeably higher activity (IC50 = 4.1 µM) and completely arrested division of treated embryos at only 8 µM. At this concentration morphological alterations similar to those induced by 2*E*,4*E*-decadienal [14] were observed: embryos remained blocked at the zygote stage and after about 90 min some blebbing vesicles became visible (not shown).

**Scheme 1:**
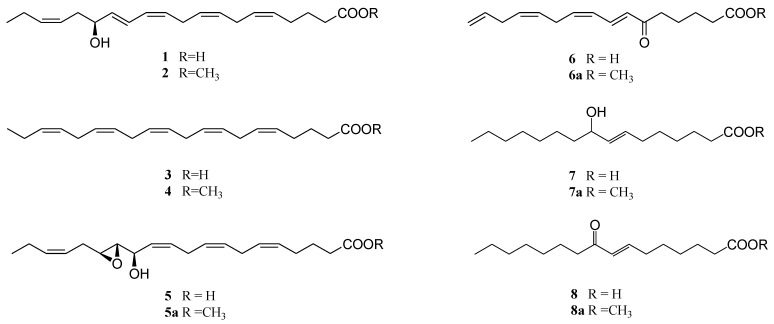
Structures of typical diatom oxylipins.

The antimitotic effect was clearly dependent on the insertion of the oxygenated function, since eicosapenataenoic acid (EPA), as both free carboxylic acid (**3**) and methyl ester (**4**), had no significant activity on sea urchin cleavage progression. Differently from the response with 15(*S*)-HEPE, the methyl ester of EPA (**4**) was fully inactive up to 330 µM (Figure 1a) whereas the free fatty acid (**3**) started interfering with the development of sea urchin embryos only at concentrations above 165 µM. At even higher levels (330 µM), this compound also blocked cleavage, but the effect was clearly distinguishable from that induced by **2** since embryos treated with high concentrations of EPA (**3**) became progressively diaphanous and after about 90 minutes started to swell and increased in volume with loss of cellular organization.

The unexpectedly potent activity of **2** prompted us to investigate other classes of diatom oxylipins. Thus, compounds (**5**–**8**) were isolated from different diatom species [1,10] and tested on sea urchin embryos. In analogy with HEPE and EPA, methyl ester derivatives **5a**–**8a** were also prepared from natural products by derivatization with diazomethane.

No activity was found with free carboxylic acids **5**–**8** at concentrations up to 100 µM (not shown) but methylation of the carboxylic function determined a generalized increase of the inhibitory activity of these compounds on development of sea urchin embryos (Figure 1). In agreement with the effect previously described with 15(*S*)-HEPE (**2**), compounds **5a** and **7a** arrested mitotic divisions at 52 and 75 µM (IC50 = 22.6 and 33.0 µM, respectively), whereas compounds **6a** and **8a** showed a stronger effect and inhibited 100% egg cleavage at 6 and 9 µM, respectively (IC50 = 4.2 and 6.9 µM). These results suggested that unsaturated hydroxy- or keto-groups are crucial to enhance the activity of these fatty acid derivatives. Interestingly, these compounds were as potent as decadienal, which is reported to fully block cleavage of sea urchin embryos at 5 µM [14,15]. Pohnert and colleagues discussed the effect of decadienal and other unsaturated-aldehydes produced by diatoms in relation to non-specific chemical reactivity towards nucleophilic biomolecules [16]. Following other studies on biologically active aldehydes, these authors suggested that the main reaction pathway was the Michael-addition of the unsaturated aldehyde with thiol or amino groups of proteins. These reactions are not specific and, consequently, the adduct formation induces adverse effects on a broad range of cell functions. Similar mechanism could be also suggested for compounds **6a** and **8a**. Conversely, the absence of activity in the analogs **6** and **8** could be due to kinetic factors including degradation processes or the inability to reach the potential targets.

### 2.2. Synthesis of Oxylipin Analogues and Evaluation of Inhibitory Activity on Development of Sea-Urchin Embryos

**Scheme 2:**
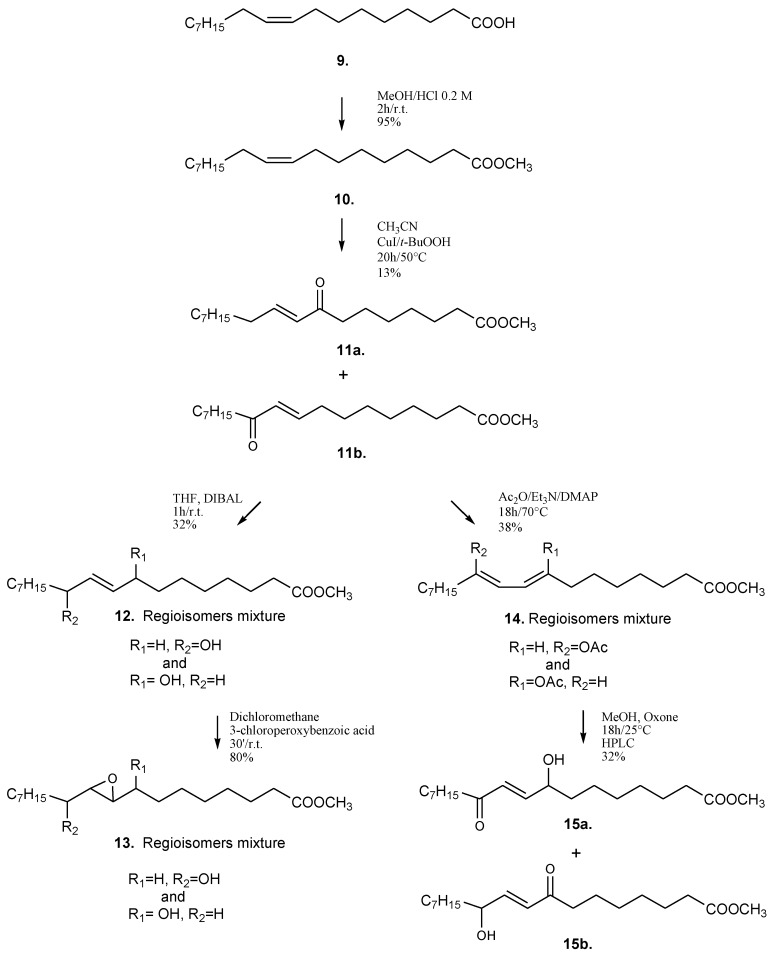
Synthetic strategy for preparation of C18-analogs of diatom oxylipins.

Considering the inherent chemical instability of the polyunsaturated chain and the biological effects of compounds **6a** and **8a**, we synthesized a number of analogs containing a rational combination of the oxygenated functions found in natural oxylipins. Preliminarily, using oleic acid as starting material, a synthetic strategy was designed in order to have access to different types of C18-compounds (Scheme 2). To this aim, allylic oxidation of oleic acid methyl ester by copper (I) iodide and *t*-butyl hydroperoxide [17] yielded the mixture of α,β-unsaturated keto regioisomers **11a** and **11b**. The following reduction by DIBAL in THF gave the regioisomers mixture **12** of enol derivatives that was further oxidized to the isomeric epoxy-alcohols **13** by 3-chloroperoxybenzoic acid (MCPBA). In another reaction, the isomeric enones **11a**/**11b** were acetylated giving rise to the corresponding enols **14** which were then oxidized at the allylic position by Oxone^®^ [18] obtaining the regioisomeric mixture **15** that was further purified giving compounds **15a** and **15b**.

**Figure 2:**
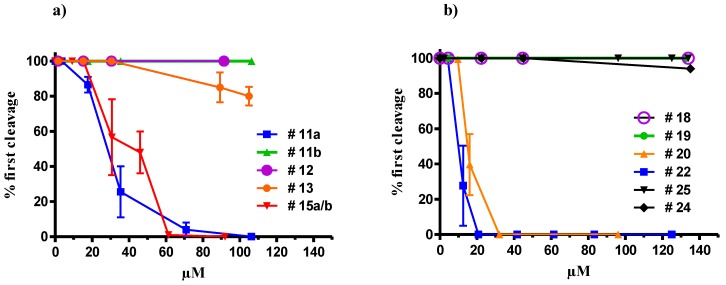
Cleavage inhibition in sea urchin embryos treated with increasing concentrations of (**a**) C_18_-fatty acid synthetic derivatives and (**b**) synthetic C_15_-alkyl derivatives. Compound numbering is according to the structure reported in Scheme 2 and Scheme 3. Values (means ± S.D.; *N* = 600) are the results of three different experiments.

All these products were assessed for their ability to interfere with the development of sea urchin embryos. In agreement with the results on natural compounds, the synthetic products containing the free carboxylic function were not active (data not shown). The α,β-unsatured keto-ester **11a** gave the best results (IC50 = 28.0 µM) together with the corresponding ketols **15a**/**15b** (IC50 = 46.0 µM) (Figure 2a).

**Scheme 3:**
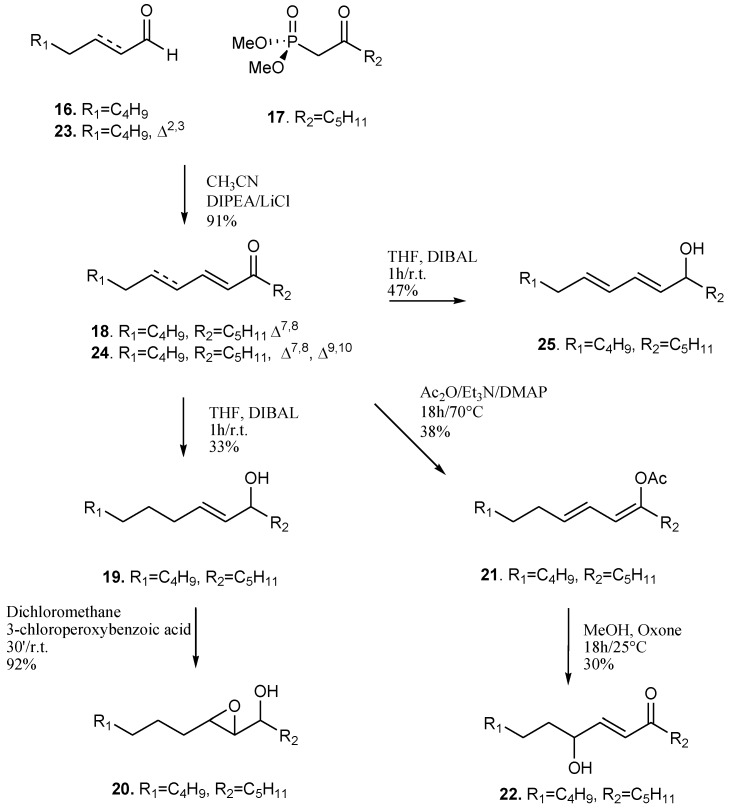
Synthetic scheme for preparation of compounds **18**–**25**.

In agreement with the results obtained with natural oxylipins, the activity of these molecules suggested that the presence of the enone moiety (Michael acceptor) is directly related to the inhibition of development of sea urchin embryos. Introduction of the hydroxy group affected the electrophilicity of the enone, thus possibly explaining the difference in potency of **11a** and the regioisomeric mixture **15a**/**15b**. Nevertheless, the significant difference between **11a** and **11b** activity after chromatographic purification, indicated that other structural features were also required to trigger the effect, thus corroborating the hypothesis that the mechanism of action of these molecules is due to interactions with specific cellular targets.

To further simplify the molecular scaffold and to increase the lipophilicity of the molecule, we prepared a second series of products without the carboxy group by using a combinatory approach that relies on olefination of two synthons by Horner-Wadsworth-Emmons reaction. The strategy is very versatile and has been used to prepare a large number of compounds starting from commercial reagents. Because the C_16_-compound **8a** was significantly more potent than the analogous C_18_-products **11a**/**11b**, we focused on the preparation of molecules with shorter alkyl chains. As reported in Scheme 3, stable C_15_-derivatives containing ketol and enone functions were easily obtained by coupling of C_8_-aldehydes and dimethyl 2-oxoheptylphosphonate (**16**) with **17**. This way, the reaction of octanal led to the α,β-keto-unsaturated compound **18** that quantitatively gave the enol derivative **19** by DIBAL reduction. This latter product could be easily transformed to the epoxyalcohol **20** by epoxydation with MCPBA. γ-Ketol **22** was obtained from **18** by acetylation and Oxone^®^ oxidation. Analogously, dienone **24** and dienol **25** were obtained starting from octenal (**23**).

Activity of compounds **18**–**22** and **24**–**25** on sea urchin embryos is reported in Figure 2b. Surprisingly, compound **18** that showed the closest analogy with the methyl ester of the natural compound **8a** (IC50 = 6 µM) was totally ineffective in inhibiting the development of the embryos. On the contrary, the epoxyalcohol **20** and the ketol **22** drastically reduced the percentage of dividing embryos. The latter compound showed an IC50 of 10 µM that was close to the value found with the natural compound **8a**.

### 2.3. Apoptotic Activity of Compound **22**

**Figure 3:**
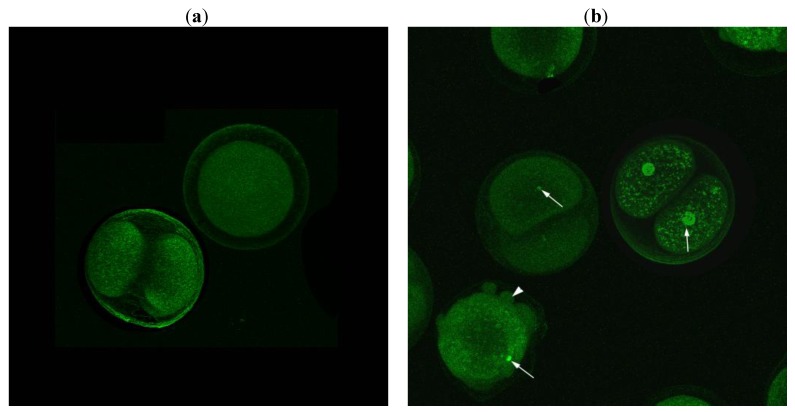
Z-projections of confocal images of sea urchin eggs stained with TUNEL 70 min after fertilization: (**a**) control and (**b**) eggs treated with 10 µM of **22**. Arrows indicate positive nuclei that appear fluorescent and arrowhead indicates blebbing on egg surface.

In analogy with the effect of exposure to high concentrations of natural oxylipins [2,14], sea urchin embryos treated with compound **22** revealed a dose-dependent increase in bleb formation. Because this effect had been previously associated with apoptosis [14], we studied the response of embryos to ketol **22** by terminal deoxynucleotidyl transferase (TdT) dUTP Nick-End Labeling (TUNEL) assay that is routinely used to detect extensive DNA degradation during the late stages of apoptosis. Confocal images of the sea urchin embryos treated with **22** at 10 µM clearly revealed that some of the nuclei were intensely fluorescent in agreement with the onset of the DNA degradation (Figure 3), as already reported with pro-apoptotic aldehydes produced by diatoms [14]. Other cells showed barely stained nuclei due to progression of DNA degradation with consequent reduction and diffusion of nuclear material. In addition, the occurrence of blebbing vesicles was clearly visible (Figure 3b, arrowhead).

**Figure 4:**
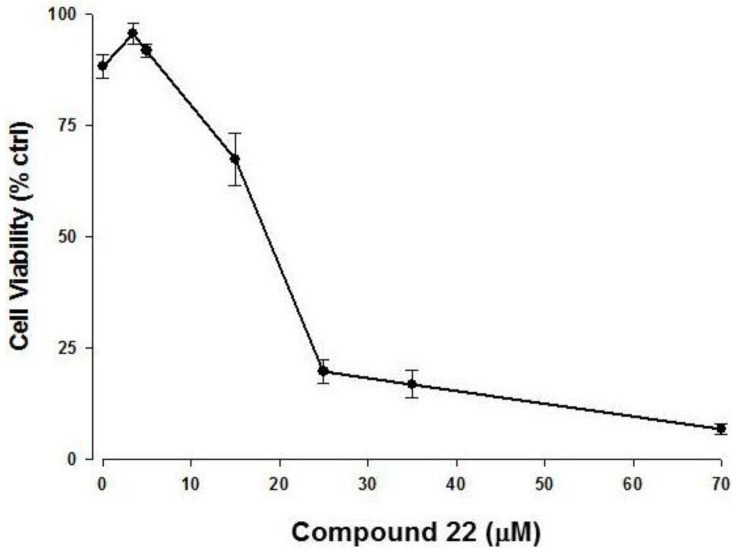
Compound **22** is cytotoxic in U-937 cell line. Cells were treated with different doses of **22**, as indicated. Cytotoxicity was measured by neutral red assay and reported as a percentage of DMSO treated control cells, as described in Methods. Data points represent the mean of three experiments (±S.E.).

To determine whether compound **22** induced apoptosis in tumor cell lines, U-937 cells were incubated with increasing concentrations of the synthetic product. This cell line was selected on the basis of its resistance to apoptotic stimuli determined in previous studies [19]. As reported in Figure 4, the ketol **22** showed a dose-dependent cytotoxicity with an apparent EC50 of about 19 µM.

**Figure 5:**
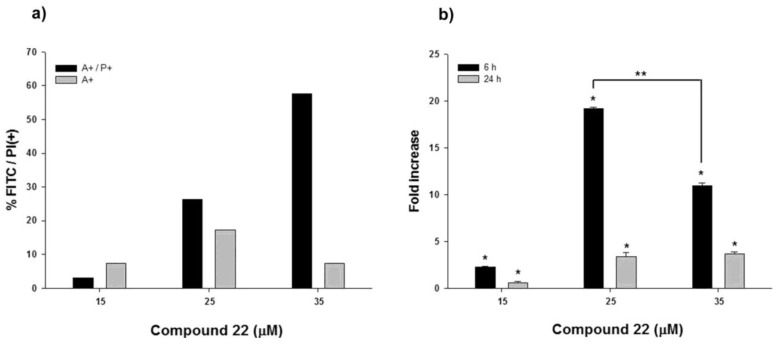
Compound **22** induces apoptosis in U-937 cell line. (**a**) Cells were treated with **22** at the concentrations indicated for 24 h. Apoptosis was quantified by Annexin V-FITC staining, measured with flow cytometry, and normalized with respect to controls treated with DMSO. Bars A+ indicate positivity to Annexin V, while bars A+/P+ indicate positivity to Annexin V and propidium iodide. (**b**) Proteolytic activities of caspases-3 was measured after 6 and 24 h and reported as -fold increase with respect to DMSO-treated cells. In panel (**b**), bars represent the mean of three experiments (±S.E.). Symbols indicate significance: *p* < 0.01 (*) with respect to DMSO-treated samples; *p* < 0.001 (**).

To determine the mechanism leading to cell death and to discriminate between induction of necrotic or apoptotic processes, cells treated with **22** were stained with Annexin-V to detect Phosphatidylserine (PS) externalization, as a marker of an active apoptotic process. At 25 µM, **22** efficiently induced apoptosis as demonstrated by the coincidence between the population of Annexin-V positive cells and the total population of dead cells (Figure 5a). Nevertheless, at higher concentrations (above 35 µM) a prevalence of a necrotic process was observed, as indicated by the high positivity to propidium iodide staining with respect to Annexin-V (Figure 5b).

The same trend was confirmed by the determination of caspase-3 activity, a hallmark of apoptotic induction in several cellular models. Compound **22** induced about a 20-fold increase in caspase-3 activity after only 6 h. After 24 h, although caspase-3 activity remained higher compared to controls, its enzymatic activity was significantly reduced in comparison with the beginning of the experiments, thus indicating the presence of secondary apoptotic events and/or necrotic cell death (Figure 5b). According to these data it is reasonable to suggest that ketol **22** induces apoptosis by activation of specific cell mechanisms (e.g., caspase-3 in leukemia–derived U-937 cells) at low concentrations, whereas it is responsible for general cytotoxicity at higher concentrations. Further studies are necessary to evaluate the pro-apoptotic activity of active diatom oxylipins that inspired the synthesis of compound **22**.

Inhibition of cell division, apoptotic and necrotic cell degradations by other Michael acceptors, such as the model short chain aldehyde 2*E*,4*E*-decadienal, have already been reported in experiments performed *in vitro* and *in vivo* [14,20,21,22]. On the contrary, studies on non-volatile oxylipin activity are still scarce. In this context, the ketol **22** could be used as a model compound to unravel the molecular mechanisms of oxylipin action in different biological systems.

## 3. Experimental Section

1D- and 2D-NMR spectra were recorded on a Bruker Avance-400 (400.13 MHz) and on a Bruker DRX-600 equipped with a TXI CryoProbe in CDCl_3_ and C_6_D_6_ (δ values are referred to CHCl_3_ and C_6_HD_5_ at 7.26 and 7.16 ppm, respectively) and ^13^C NMR spectra were recorded on a Bruker DPX-300 (75.47 MHz) (δ values are referred to CDCl_3_ and C_6_D_6_ at 77.0 and 128.0 ppm respectively). HRESI^+^-MS was measured on a Micromass Q-TOF micro. TLC plates (Kieselgel 60 F_254_) and silica gel powder (Kieselgel 60, 0.063–0.200 mm) were from Merck (Merck and Co., Darmstadt, Germany).

All the reagents were purchased from Sigma-Aldrich (Milan, Italy) and used without any further purification.

All the synthetic intermediates were characterized by MS and NMR analysis.

### 3.1. Chemical Synthesis of Oxylipin Analogues

**Compounds 11a and 11b**: Following the procedure of Salvador *et al.* [17] oleic acid methylester (**10**, 1g, 3.5 mmol) was dissolved in acetonitrile (18 mL) under argon; copper (I) iodide (6.8 mg, 0.035 mmol) and t-butylhydroperoxide (4.16 mL, 23 mmol) were added; the reaction mixture was stirred at 50 °C for 20 h. Subsequently the solution was poured into sodium sulfite solution (10% aq.) and extracted with diethyl ether; the extract was washed with aq. saturated solution of NaHCO_3_, brine and water, dried (MgSO_4_), evaporated to dryness and was purified by silica-gel column using a gradient of petroleum ether and diethylether to give **11** as a mixture of regioisomers (140 mg, 0.45 mmol, 13%); R*_f_* (Petroleum ether/diethyl ether 7:3) = 0.53; ^1^H-NMR (400 MHz, C_6_D_6_): δ = 6.66 (dt, *J* = 15.7, 6.4 Hz, 1H), 6.01 (bd, *J* = 15.7 Hz, 1H), 3.38 (s, 3H; OCH_3_), 2.29 (t, *J* = 7.4, 2H), 2.08 (t, *J* = 7.4 Hz, 2H; H_2_2), 1.81 (m, 2H), 1.54 (m, 2H), 1.32-1.15 (m, 20H), 0.89 (t, *J* = 6.5, 3H; H_3_18); UV λ_max_ (MeOH) 219 nm (ε = 4401); HRMS (ESI^+^): *m/z* calcd for C_19_H_34_O_3_Na: 333.2406; found: 333.2411; part of mixture **11** (10 mg) was purified on direct-phase HPLC (*n*-hexane/AcOEt 92/8, flow rate 2 mL/min) to afford **11a** (4 mg) and **11b** (3 mg). The compounds were characterized by NMR, and in particular 2D-TOCSY, was diagnostic to distinguish two spin systems.

**Mixture 12**: mixture **11** (70 mg, 0.23 mmol) was dissolved in fresh distilled THF (1.0 mL) and 0.17 mL of diisobutylaluminium hydride solution in THF (1M) were drop-wise added at 0 °C and the mixture was stirred at room temperature for 1 h and after was partitioned between water and diethylether; the organic phase was purified by silica-gel column using a gradient of petroleum ether and diethylether to give **12** (22 mg, 0.074 mmol, 32%); R*_f_* (Petroleum ether/diethyl ether 7:3) = 0.22; ^1^H-NMR (400 MHz, C_6_D_6_): δ = 5.50 (dd, *J* = 15.9, 6.0 Hz, 1H), 5.44 (dt, *J* = 15.9, 5.5 Hz, 1H), 3.94 (m, 1H), 3.38 (s, 3H,OCH_3_), 2.10 (t, *J* = 7.1 Hz, 2H; H_2_2), 1.95 (m, 2H), 1.69–1.25 (m, 22H), 0.90 (t, *J* = 6.6, 3H; H_3_18). HRMS (ESI^+^): *m/z* calculated for C_19_H_36_O_3_Na: 335.2512; found: 335.2517.

**Mixture 13**: mixture **12** (20 mg, 0.067 mmol) was dissolved in anhydrous dichloromethane (5 mL) and 150 mg of 3-chloroperoxybenzoic acid (MCPBA, purity ≤ 77%) dissolved in 3 mL of anhydrous dichloromethane was added drop-wise at 0 °C; the mixture was stirred for 30 min and purified by silica-gel column using a gradient of petroleum ether and diethylether to give **13** as a mixture of regioisomers (18 mg, 0.054 mmol, 80%); R*_f_* (Petroleum ether/diethyl ether 6/4) = 0.1; ^1^H-NMR (400 MHz, C_6_D_6_): δ = 3.36 (s, 3H; OCH_3_), 3.29 (m, 1H), 2.73 (m, 1H), 2.54(m, 1H), 2.09 (t, *J* = 7.5 Hz, 2H; H_2_2), 1.73–1.20 (m, 24H), 0.90 (t, *J* = 6.2, 3H; H_3_18). HRMS (ESI^+^): *m/z* calcd for C_19_H_36_O_4_Na: 351.2512; found: 351.2507.

**Mixture of Compounds 15a and 15b**: Mixture **11** (70 mg, 0.23 mmol), *N*,*N*-dimethylaminopyridine (3.1 mg, 0.025 mmol), triethylamine (0.051 g, 0.51 mmol) and acetic anhydride (0.126 g, 1.26 mmol) were stirred for 18 h at 70 °C; the mixture was partitioned between diethylether and ammonium bicarbonate (10%) and the organic phase was purified by silica-gel column using a gradient of petroleum ether/diethylether to obtain the acetate intermediate **14** [18], that was dissolved in methanol (0.5 mL) and sodium bicarbonate (saturated) buffer solution (0.5 mL) of Oxone^®^ (0.052 mmol); after stirring at 25 °C for 7 h, methanol (0.5 mL), water (0.5 mL) and oxone (0.052 mmol) were added and the mixture was kept under stirring overnight; after partition between water and chloroform, the organic phase was purified by silica-gel column using a gradient of petroleum ether and diethylether to give **15** as a mixture of regioisomers (9 mg, 0.0276 mmol, 12%); R*_f_* (Petroleum ether/diethyl ether 6/4) = 0.1; ^1^H-NMR (400 MHz, C_6_D_6_): δ = 6.64 (dd, *J* = 15.7, 4.5 Hz, 1H), 6.23 (dd, *J* = 15.7, 1.4 Hz, 1H), 3.79 (m, 1H), 3.39 (s, 3H; OCH_3_), 2.28 (t, *J* = 7.2 Hz, 2H), 2.06 (t, *J* = 7.2 Hz, 2H; H2), 1.69 (m, 2H), 1.51 (m, 2H), 1.30-1.11 (m, 16H), 0.89 (t, *J* = 6.2, 3H; H_3_18); ^13^C-NMR (100MHz, C_6_D_6_): δ = 199.2 (C), 147.2 (CH), 128.0 (CH), 70.9 (CH), 51.0 (OCH_3_), 41.03 (CH_2_), 36.7 (CH_2_), 33.9 (CH_2_), 32.1 (CH_2_), 30.3 (CH_2_), 29.8 (CH_2_), 29.6 (CH_2_), 29.1 (CH_2_), 25.1 (2 CH_2_), 24.4 (CH_2_), 23.0 (CH_2_), 14.3 (CH_3_). UV λ_max_ (MeOH) 219 nm (ε = 1570); HRMS (ESI^+^): *m/z* calcd for C_19_H_34_O_4_Na: 349.2355; found: 349.2359. Mixture **15** was purified on reverse-phase HPLC (methanol/water gradient 6/4 until 9/1, flow rate 2.5 mL/min) to afford **15a** (3 mg) and **15b** (5 mg).

**Compound 18**: Dimethyl 2-oxoheptylphosphonate (**17**, 2.67 g, 12 mmol), *N*,*N*-diisopropylethylamine (1.29 g, 10 mmol) and octanal (**16**, 1.28 g, 10 mmol) were added to a solution of lithium chloride (0.50 g, 12 mmol) in acetonitrile (120 mL) under argon; after stirring for 3 h the reaction mixture was evaporated and purified by silica-gel column using a gradient of petroleum ether/diethylether gradient to give **18** (2.01 g, 9.1 mmol, 91%) [23]: R*_f_* (Petroleum ether/diethyl ether 95:5) = 0.52; ^1^H-NMR (400 MHz, C_6_D_6_): δ = 6.62 (dt, *J* = 15.7, 6.7 Hz, 1H; H6), 5.93 (bd, *J* = 15.7 Hz, 1H; H7), 2.29 (t, *J* = 7.4, 2H; H_2_9), 1.99 (m, 2H; H_2_5), 1.52 (m, 2H), 1.30–1.15 (m, 14H), 0.81 (t, *J* = 6.2, 6H; H_3_1, H_3_15 ); ^13^C-NMR (100MHz, C_6_D_6_): δ = 198.3 (C8, C), 145.9 (C6, CH), 130.8 (C7, CH), 40.1 (C9, CH_2_), 32.6 (CH_2_), 32.1 (2 CH_2_), 29.6 (2 CH_2_), 29.5 (CH_2_), 24.2 (CH_2_), 23.0 (2 CH_2_), 14.3 (2 CH_3_). HRMS (ESI^+^): *m/z* calcd for C_15_H_28_ONa: 247.2038; found: 247.2033.

**Compound 19**: Ketone **18** (0.06 g, 0.25 mmol) was dissolved in fresh distilled tetrahydrofurane (1 mL) and 0.150 mL of diisobutylaluminium hydride solution in THF (1 M) were drop-wise added at −5 °C; after 1 h of stirring, other 0.015 mL of DIBAL solution in THF (1 M) were added; after 1 h the mixture was partitioned between water and diethylether; the organic phase was purified by silica-gel column using a gradient of petroleum ether and diethylether to give **19** (0.02 g, 0.08 mmol, 33%); R*_f_* (Petroleum ether/diethyl ether 95:5) = 0.12; ^1^H-NMR (400 MHz, C_6_D_6_): δ = 5.54 (dt, *J* = 15.5, 6.4 Hz, 1H; H8), 5.43 (dd, *J* = 15.5, 6.2 Hz, 1H; H7), 3.94 (m, 1H; H6), 1.99 (m, 2H; H_2_9), 1.55–1.20. (m, 18H), 0.89 (t, *J* = 6.4, 6H; H_3_1, H_3_15 ); ^13^C-NMR (100MHz, C_6_D_6_): δ = 134.2 (CH), 131.2 (CH), 73.0 (C6, CH) 37.9 (CH_2_), 32.6 (CH_2_), 32.3 (2 CH_2_), 30.0 (CH_2_), 29.8 (CH_2_), 29.5 (CH_2_), 25.6 (CH_2_), 23.1 (2 CH_2_), 14.3 (2 CH_3_).

UV λ_max_ (MeOH) 220 nm (ε = 1295); HRMS (ESI): *m/z* calcd for C_15_H_30_ONa: 249.2195; found: 249.2199.

**Compound 20**: Alcohol **19** (20 mg, 0.08 mmol) was dissolved in anhydrous dichloromethane (1.5 mL) and 3-chloroperoxybenzoic acid (50 mg) dissolved in 1 mL of anhydrous dichloromethane was added drop-wise at 0 °C; the mixture was stirred for 30 min and purified by silica-gel column using a gradient of petroleum ether and diethylether to give **20** (18 mg, 0.074 mmol, 92%); R*_f_* (Petroleum ether/diethyl ether 8/2) = 0.25; ^1^H-NMR (400 MHz, C_6_D_6_): δ = 3.29 (m, 1H), 2.73 (m, 1H), 2.54 (m, 1H), 1.62-1.20 (m, 20H), 0.88 (t, *J* = 6.7, 6H; H_3_1, H_3_15). HRMS (ESI^+^): *m/z* calcd for C_15_H_30_O_2_Na: 265.2144; found: 265.2141.

**Compound 22**: Ketone **18** (0.97 g, 4.4 mmol), *N*,*N*-dimethylaminopyridine (58 mg, 0.48 mmol), triethylamine (0.98 g, 9.7 mmol) and acetic anhydride (2.4 g, 24 mmol) were stirred for 18 h at 70 °C; the mixture was partitioned between diethylether and ammonium bicarbonate (10%) and the organic phase was purified by silica-gel column using a gradient of petroleum ether/diethylether to obtain the acetate intermediate **21**, (0.44 g, 1.654 mmol) that was dissolved in methanol (3 mL) and sodium bicarbonate (saturated) buffer solution (2 mL) of oxone (1 mmol); after stirring at 25 °C for 7 h, methanol (2 mL), water (2 mL) and oxone (1 mmol) were added and the mixture was kept under stirring overnight; after partition between water and chloroform, the organic phase was purified by silica-gel column using a gradient of petroleum ether and diethylether to give **22** (0.120 g, 0.5 mmol, 10%) [18]. R*_f_* (Petroleum ether/diethyl ether 95:5) = 0.1; ^1^H-NMR (400 MHz, C_6_D_6_): δ = 6.68 (dd, *J* = 15.6, 5.3 Hz, 1H; H8), 6.25 (dd, *J* = 15.6, 1.2 Hz, 1H; H7), 3.86 (m, 1H; H9), 2.25 (t, *J* = 7.2 Hz, 2H; H_2_5), 1.62 (m, 2H), 1.35-1.18 (m, 16H), 0.90 (t, *J* = 6.4, 6H; H_3_1, H_3_15 ); ^13^C-NMR (100MHz, C_6_D_6_): δ = 199.0 (C6, C), 147.6 (C8, CH), 128.4 (C7, CH), 71.1 (C9, CH) 40.9 (C5, CH_2_), 37.0 (CH_2_), 32.0 (CH_2_), 31.7 (CH_2_), 29.5 (CH_2_), 25.5 (CH_2_), 24.0 (CH_2_), 22.9 (2 CH_2_), 14.2 (2 CH_3_).

UV λ_max_ (MeOH) 219 nm (ε = 12437); HRMS (ESI^+^): *m/z* calcd for C_15_H_28_O_2_Na: 263.1987; found: 263.1982.

**Compound 24**: Starting from *trans*-2-octenal (**23**, 1 g, 8 mmol) and following the same procedure to get **18**, compound **24** (1.60 g, 7.2 mmol, 91%) was obtained; R*_f_* (Petroleum ether/diethyl ether 95:5) = 0.42; ^1^H-NMR (400 MHz, C_6_D_6_): δ = 7.10 (dd, *J* = 15.5, 10.6 Hz, 1H; H8), 5.99 (d, *J* = 15.5 Hz, 1H; H7), 5.96 (dd, *J* = 15.3, 10.6 Hz, 1H; H9), 5.82 (dt, *J* = 15.3, 6.9 Hz, 1H; H10), 2.31 (t, *J* = 7.2 Hz, 2H; H_2_5), 1.94 (m, 2H; H_2_11), 1.61 (m, 2H), 1.29–1.13 (m, 10H), 0.88 (t, *J* = 6.8 Hz, 6H; H_3_15, H_3_1); ^13^C-NMR (100MHz, C_6_D_6_): δ = 198.8 (C6, C), 144.6 (CH), 142.1 (CH), 129.5 (CH), 128.6 (CH), 40.9 (CH_2_), 33.3 (CH_2_), 31.9 (CH_2_), 31.7 (CH_2_), 28.8 (CH_2_), 24.3 (CH_2_), 22.9 (CH_2_), 22.8 (CH_2_), 14.2 (CH_3_), 14.2 (CH_3_).

UV λ_max_ (MeOH) 264 nm (ε = 1469); HRMS (ESI^+^): *m/z* calcd for C_15_H_26_ONa: 245.1881; found: 245.1883.

**Compound 25**: Unsaturated ketone **24** (0.80 g, 3.6 mmol) was dissolved in fresh distilled THF (8.0 mL) and and 2.3 mL of diisobutylaluminium hydride solution in THF (1 M) were added drop-wise at −5 °C; the mixture was stirred at room temperature for 1 h and after was partitioned between water and diethylether; the organic phase was purified by silica-gel column using a gradient of petroleum ether and diethylether to give **25** (0.39 g, 1.7 mmol, 47%); R*_f_* (Petroleum ether/diethyl ether 95:5) = 0.16; ^1^H-NMR (400 MHz, C_6_D_6_): δ = 6.23–6.00 (m, 2H), 5.69–5.00 (m, 2H), 3.95 (m, 1H; H6), 2.00 (m, 2H; H_2_11), 1.56-1.22 (m, 14H), 0.87 (t, *J* = 6.8 Hz, 6H; H_3_1, H_3_15); ^13^C-NMR (100MHz, C_6_D_6_): δ = 134.8 (CH), 134.7 (CH), 130.8 (CH), 72.8 (C6, CH), 37.8 (CH_2_), 30.1 (CH_2_), 32.4 (2 CH_2_), 29.8 (2 CH_2_), 23.2 (2 CH_2_), 14.3 (2 CH_3_).

UV λ_max_ (MeOH) 230 nm (ε = 9043); HRMS (ESI^+^): *m/z* calcd for C_15_H_28_ONa: 247.2038; found: 247.2032.

### 3.2. Sea Urchin Gamete Collection

Sea urchins *Paracentrotus lividus* (Lamarck) were collected during the breeding season by SCUBA diving in the Gulf of Naples and transported in an insulated box to the laboratory within one hour. Living organisms were injected with 0.2 mL of 0.2 M acetylcholine (Sigma-Aldrich, Milan, Italy) to induce gamete ejection. Eggs were obtained from at least four females collected separately in beakers containing 0.22 µm filtered sea water (FSW) in which they were allowed to settle. Egg suspensions were then washed three times and diluted with FSW to a final concentration of 3000 eggs mL^−1^. Sperm were collected dry as a mix from three males, stocked at 4 °C and diluted 1:10,000 just prior to fertilization. One hundred microliters of sperm suspension were added to 100 mL FSW containing egg suspension. Five minutes after sperm addition, the eggs were checked for successful fertilization and excess sperm were removed by washing the eggs with FSW.

### 3.3. Antimitotic Assay on Sea Urchin Embryos

Oxylipins were tested on sea urchin embryos assessing the effect on cell division (cleavage). Approximately 400 embryos were transferred soon after fertilization envelope elevation to tissue culture wells containing increasing concentrations of each oxylipin in 4 mL FSW. All concentrations were tested at least on three egg clutches collected from different females. Additional group of eggs from each female were used as controls. Embryos were kept at 20 °C in a controlled temperature chamber under 12:12 light:dark cycle. Effect of molecules on the first mitotic division was assessed circa 90 minutes after fertilization, when almost 100% of control embryos were at the two-blastomere stage. Cleavage inhibition was assessed by examining at least 200 embryos for each treatment under a light microscope (Axiovert 135TV, Carl Zeiss, Jena, Germany). Graphics from the experimental data and IC50 values were generated using the Prism software (GraphPad Software Inc., La Jolla, CA, USA).

### 3.4. Tunel Fluorescence Labeling (TUNEL)

Fertilization occurred in FSW with 1 mmol L^−1^ ATA (3-amino-1,2,4-triazole; Sigma-Aldrich, Milan, Italy) for immuno-fluorescence staining [14].

*P. lividus* embryos were fertilized in the presence of 1 mmol L^−1^ ATA (3-amino-1,2,4-triazole; Sigma-Aldrich, Milan, Italy) before incubation (see above) with **22** at 10 µM. After 90 min sea urchin embryos and controls were gently forced through a Pasteur pipette, to remove the fertilization envelop, rinsed three times in FSW, and fixed in paraformaldehyde 4% (Sigma-Aldrich, Milan, Italy) in sea water for 2 h at room temperature. Fixed sea urchin embryos were washed several times in 10 mM phosphate saline buffer at pH 7.4 (PBS) to remove paraformaldehyde and then incubated for 30 min, at 4 °C, in a solution of 0.1% Triton X-100 and 0.1% sodium citrate. Apoptosis was assessed using TdT-mediated dUTP nick end labeling (TUNEL) (Roche Diagnostics, Milano, Italy) according to manufacturer’s instructions. After washing in PBS containing 1% bovine albumin serum (BSA, Sigma-Aldrich, Milano, Italy), treated samples were incubated at 37 °C for 90 min in TUNEL solution in a humidified chamber, in the dark. Control embryos were fixed and stained as described above. Whole-mount sea urchin embryos were observed with an inverted confocal laser scanning microscope (CLSM) Zeiss-510 equipped with a 25× water immersion objective. Each image was acquired with an Argon 488 nm wavelength (λ) laser to detect TUNEL fluorescence (green).

### 3.5. Cell Culture and Cell Viability Assay

The human myelomonocytic cell line U937 was cultured in Roswell Park Medium Institute (RPMI) medium supplemented with 10%, fetal bovine serum and 1% penicillin/streptomycin at 37 °C in a humidified atmosphere containing 5% CO2. Cell viability assay was performed using neutral red viability test as described in [24]. RPMI medium, l-glutamine 200 mM, penicillin 5000 IU/mL/streptomycin 5000 µg/mL and PBS (phosphate buffer saline) tablets were purchased from Life Technologies (Monza, Italy); fetal bovine serum from Cambrex (Milano, Italy). Neutral red solution (0.33% v/v), trypan blue solution (0.4% v/v), propidium iodide were from Sigma-Aldrich (Milano, Italy).

### 3.6. Annexin V Assay

Phosphatidylserine (PS) exposure was measured using the binding of fluorescein-isothiocyanate-labeled (FITC) Annexin-V to PS, as indicated in the manufacturer’s protocol (Enzo Life Sciences; Vinci, Florence, Italy). Briefly, cells (2 × 10^6^ per mL) were incubated in the presence of the tested compounds for the indicated times. Cells were collected and centrifuged at 400× *g* for 5 min, washed in PBS and suspended in binding buffer (10 mM HEPES, pH 7.4; 140 mM NaCl; 2.5 mM CaCl_2_). Annexin V FITC (2 µL) and propidium iodide (25 µg/mL) were added for 10 min in the dark, at room temperature and analyzed with flow cytometer (FACS-Calibur; Becton Dickinson, Mountain View, CA, USA) equipped with argon laser (488 nm) and 530 and 585 nm filters for FITC and phycoerythrin, respectively. Low fluorescence debris and necrotic cells, permeable to propidium iodide, were gated out before analysis. Data were analyzed using CellQuest software (Becton Dickinson, Milano, Italy).

### 3.7. Caspase Assay

To determine caspase-3 enzymatic activity, 2 × 10^6^ cells were incubated in the presence of the tested compounds for 6 or 24 h. Cells were collected and centrifuged at 400× *g* for 5 min, washed twice in PBS and suspended in lysis buffer (10 mM HEPES, pH 7.4; 2 mM ethylenediaminetetracetic acid; 0.1% [3-(3-cholamidopropyl) dimethylammonio]-1-propanesulfonate; 5 mM dithiothreitol; 1 mM phenylmethylsulfonyl fluoride; 10 µg/mL pepstatin-A; 10 µg/mL apronitin; 20 µg/mL leupeptin). Cell extracts (10 µg) were added to reaction buffer containing the conjugated amino-4-trifluoromethyl coumarin (AFC) substrates: benzyloxycarbonyl-Asp(OMe)-Glu(OMe)-Val-Asp(OMe)-AFC (ZDEVD-AFC). Samples were incubated at 37 °C for 30 min. Upon proteolytic cleavage of the caspase-3 substrate, the free fluorochrome AFC was detected by a spectrofluorometer multiplate reader (FL-500; Bio-Tek Instruments, Milano, Italy) with excitation 395 ± 20 nm and emission at 530 ± 20 nm. To quantify enzymatic activity, we calculated an AFC standard curve. Caspase-3 specific activity was determined as nmol of AFC produced per min per µg proteins at 37 °C using saturating substrate concentrations (50 µM) [24]. Fold increase in caspase-3 was determined by direct comparison with the level of 0.1% DMSO-treated cells.

## 4. Conclusions

Biological organisms benefit from the ability to develop and use chemicals to communicate with each other, to defend themselves from predators and pathogens, to prey and to mate. Apart from their ecological and physiological role, these products have a promising potential as evolutionary preselected lead structures for biotechnological applications. It is therefore not surprising that in this work eco-physiological considerations on diatom oxylipins are demonstrated to be predictive for the synthesis of a novel compound with apoptotic activity against tumor cell lines. Although compound **22**, as it is, is not sufficiently potent to suggest further evaluation for antitumor activity, its chemical structure is easy to modify in order to improve pharmacological and biological activity. In this sense, the present study supports the possibility of replacing expensive high-throughput screening platforms for drug discovery using chemical synthesis inspired by bioactive secondary metabolites.

## Abbreviations

DIBAL: diisobutylaluminum hydride
THF: tetrahydrofuran
